# The Hugo™ RAS system in gynecologic robotic surgery: a systematic review of current applications

**DOI:** 10.1007/s11701-025-02973-3

**Published:** 2025-11-17

**Authors:** Chiara Innocenzi, Massimo Criscione, Matteo Pavone, Nicolò Bizzarri, Andrea Rosati, Marco Petrillo, Riccardo Oliva, Denis Querleu, Anna Fagotti, Lise Lecointre, Mariano Giménez, Barbara Seeliger, Antonello Forgione, Francesco Fanfani, Jacques Marescaux

**Affiliations:** 1 UOC Ginecologia Oncologica, Dipartimento di Scienze per la salute della Donna e del Bambino e di Sanità Pubblica, Fondazione Policlinico Universitario A. Gemelli, IRCCS, Rome, Italy; 2 Research Institute against Digestive Cancer, IRCAD, Strasbourg, France; 3 Università Cattolica del Sacro Cuore, Rome, Italy; 4 Department of Medicine, Surgery and Pharmacy, Gynecologic and Obstetric Clinic, University of Sassari, Sassari, Italy; 5 Institute of Image-Guided Surgery, IHU Strasbourg, Strasbourg, France; 6 ICube, UMR 7357, CNRS, INSERM U1328 RODIN, University of Strasbourg, Strasbourg, France; 7 Department of Gynecologic Surgery, University Hospitals of Strasbourg, Strasbourg, France; 8 Division of Minimally Invasive Surgery, DAICIM Foundation, Buenos Aires, Argentina; 9 Department of Digestive and Endocrine Surgery, University Hospitals of Strasbourg, Strasbourg, France

**Keywords:** Minimally invasive surgery, Robotic gynecologic surgery, Surgical innovation, Digital surgery

## Abstract

**Introduction:**

The Hugo™ Robotic-Assisted Surgery (RAS) system is a recent development in minimally invasive gynecologic surgery. Following regulatory approval, its clinical use has expanded. However, comprehensive evidence on feasibility, safety and surgical outcomes has not been established. This systematic review synthesizes the available clinical data on the application of this robotic system across gynecologic procedures.

**Materials and methods:**

A systematic review was conducted in accordance with PRISMA guidelines and registered in PROSPERO (CRD42024519329), interrogating PubMed, Web of Science and Embase databases. Manuscripts reporting data on patients undergoing gynecologic surgical procedures with the Hugo™ RAS platform were included in the analysis.

**Results:**

Qualitative analysis was conducted on twelve studies including 445 patients. Hysterectomy was the main procedure (either total or supracervical; *n* = 289, 64.9%), with primary indications including fibromatosis (*n* = 149, 33.5%) and endometriosis (*n* = 92, 20.7%). Some studies (*n* = 5, 41.7%) included patients with gynecologic malignancies, mainly endometrial cancer; one reported robotic management of a perivesical recurrence. Few conversions (laparotomy *n* = 1, 0.22%; laparoscopy *n* = 5, 1.12%), intraoperative complications (*n* = 4, 0.90%), and postoperative complications (Clavien-Dindo grade ≥ II, 1.57%) occurred. Weighted mean operative, console and docking times were 141.84 ± 28.73, 97.98 ± 28.13, and 5.90 ± 0.94 min, respectively. Estimated blood loss averaged 110.75 ± 84.16 mL, and mean hospital stay was 2 ± 1 days. Reported system malfunctions were generally manageable without significant clinical consequences.

**Conclusions:**

Current evidence indicates that the system can be safely implemented by gynecologic surgeons in routine clinical practice. Nevertheless, further high-quality research is required to determine long-term outcomes and to assess its integration within the evolving landscape of digital surgical technologies.

**Supplementary Information:**

The online version contains supplementary material available at 10.1007/s11701-025-02973-3.

## Introduction

Although the conceptual foundations of robotics emerged in the early 20th century, the translation of these principles into surgical practice did not occur until much later [1]. Robotic-assisted surgery (RAS) is now firmly established across multiple surgical disciplines, with Operation Lindbergh in 2001 representing a landmark in remote robotic laparoscopic surgery and laying the foundation for telesurgery [2]. Technological progress now advances at an unprecedented pace, driving the integration of artificial intelligence and automation, which are steadily evolving from prospective concepts to tangible clinical realities [3]. In gynecology, just as laparoscopy once replaced laparotomy, robot-assisted surgery is increasingly adopted, notably in complex cases such as gynecologic cancer recurrences [4], deep infiltrating endometriosis [5] and challenging urogynecologic procedures [6], where the surgeon’s enhanced dexterity and the instrument’s greater range of motion mitigate some of the technical or anatomical challenges intrinsic to conventional laparoscopy.

The da Vinci surgical robotic system set the standard for efficacy and safety [7], until the expiration of Intuitive Surgical’s patents in 2019, which sparked a wave of new robotic platforms focused on improving cost-effectiveness and expanding surgical capabilities [7]. In this landscape, the last robotic surgical system introduced by Medtronic (Minneapolis, MN, United States) was the Hugo™ RAS, characterized by a modular design made up of independent robotic arms, an open 3D-HD console and a system tower integrating commercially available camera and electrosurgery units. The adoption of RAS is expected to expand further, driven also by the integration of digital interfaces and embedded technologies to facilitate real-time connectivity [8].

Since its approval for clinical use in gynecology in 2021 and following the first European procedure performed the same year [9], the Hugo™ RAS has been progressively adopted in clinical practice, with several experiences subsequently documented in the literature. However, comprehensive evidence regarding the use of Hugo™ RAS system in gynecologic surgery remains limited.

This systematic review aims to evaluate and synthetize the available clinical evidence of the Hugo™ RAS system across multiple gynecologic procedures.

## Methods

The comprehensive review followed the guidelines outlined in the Preferred Reporting Items for Systematic Reviews and Meta-Analyses (PRISMA) [10] and was officially registered with the International Prospective Register of Systematic Reviews (PROSPERO, registration number CRD42024519329) prior to data extraction. Relevant articles were identified through systematic searches of the PubMed, Web of Science and Embase databases until 8th February 2025 following the PICO strategy (Patient, Intervention, Comparison and Outcome) and using the following search terms: “Hugo™ RAS”, “robotic” or “robotics”, “gynecology”, “gynecologic surgery”, “gynecologic surgical procedures”. Searches were limited to English-language articles, without restriction on initial publication date or study design. The complete search strategy is reported in the supplementary material (Online Supplement A).

### Data extraction and outcomes measurements

**Fig. 1:**
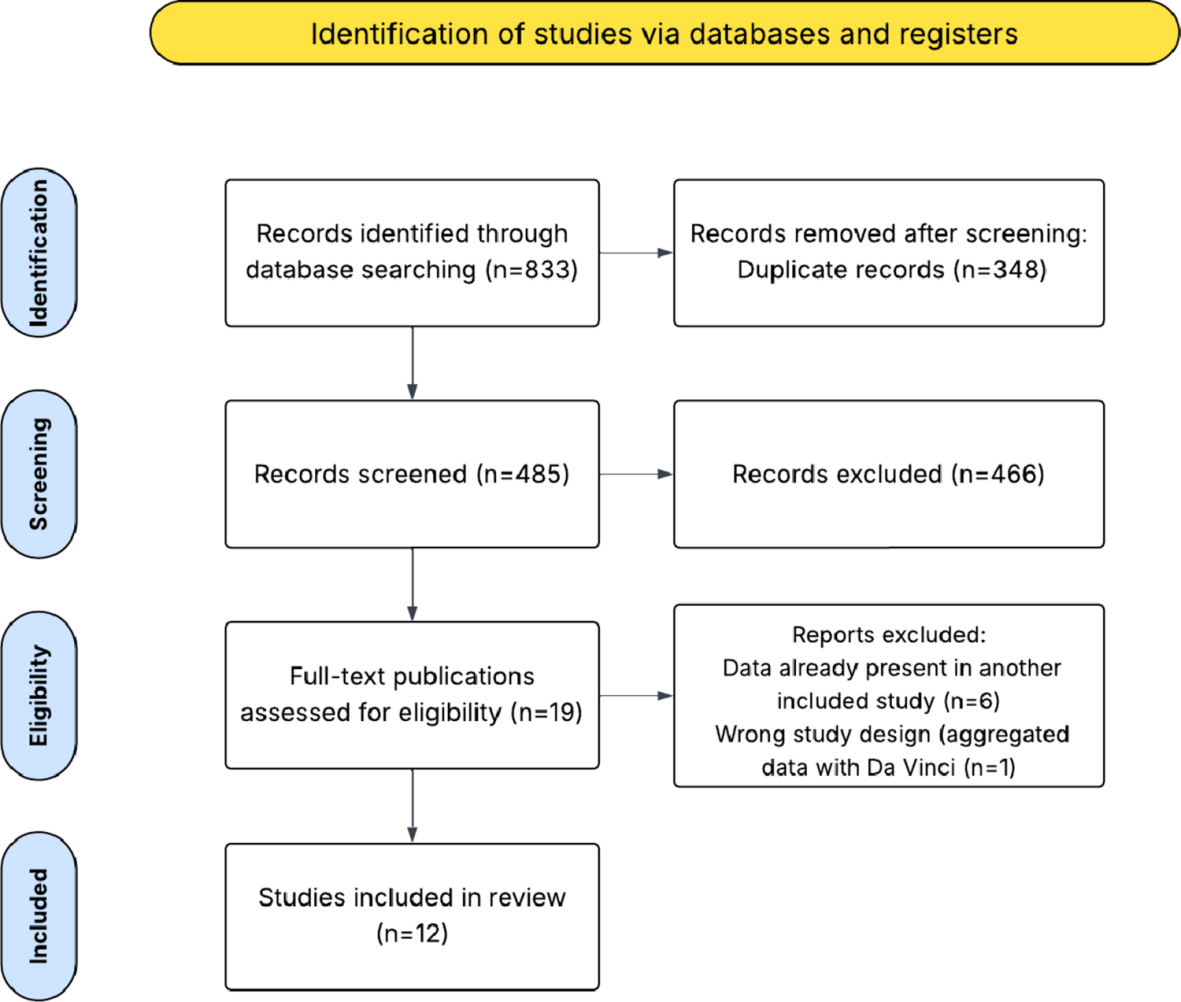
PRISMA flow diagram of selected studies

Two authors (M.C. and C.I.) independently screened titles and abstracts for eligibility using Rayyan software (Qatar Computing Research Institute, HBKU, Doha, Qatar) [11]. After removing duplicate publications, titles and abstracts were independently reviewed for inclusion, followed by a full-text assessment of eligible articles. Any discrepancies were resolved through consensus with a third author (M.P.). The inclusion criterion was the application of the Hugo™ RAS system in the field of gynecology. Abstracts, reviews, meta-analyses and editorials were excluded. The PRISMA diagram shows the complete review process from the original search to the final selection (Fig. 1). Excluded articles are reported in the supplementary material (Online Supplement B).

**Fig. 2:**
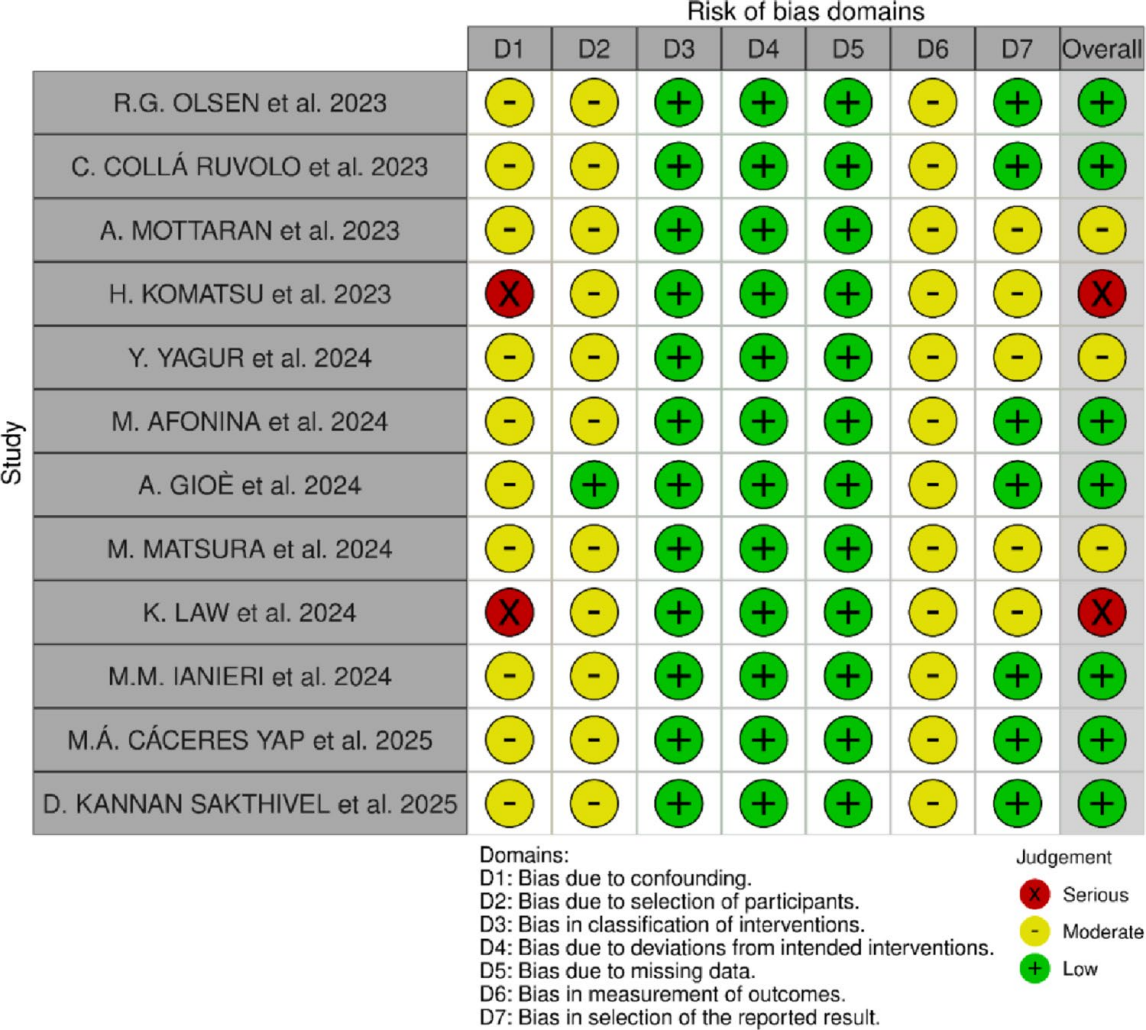
Risk of bias assessment according to the ROBINS-I tool (14)

The primary outcome of this study was to assess the feasibility and safety of the Hugo™ RAS system in gynecologic surgery by evaluating the conversion rate to laparotomy or laparoscopy along with associated intraoperative and postoperative complications. Feasibility was defined by the maintenance of the initial surgical plan. Safety was assessed by the absence of serious adverse events related to the device [12]. Collected patient data included age, body mass index (BMI), history of prior abdominal surgery, comorbidities, indication for surgery, type of procedure performed, estimated blood loss, docking time, operative time and length of hospital stay. Docking time was defined as the interval from positioning the robotic patient cart to the insertion of robotic instruments through the ports, whereas operative time was measured from the initial skin incision to final skin closure. The Clavien-Dindo classification system was used to grade intraoperative and postoperative complications [13]. Two reviewers (M.P. and M.C.) independently assessed the risk of bias using the ROBINS-I tool [14], summarized in Fig. 2.

A qualitative data analysis was chosen for the relatively low number of available studies, with weighted means used to represent the varying cohort sizes, and representation as means and standard deviations to facilitate comparability. Weighted means were calculated with each study weighted by sample size. These values are descriptive and not intended for inferential statistical conclusions. If necessary, standard deviations were derived from reported study means, and mean and standard deviation were estimated from reported median and range or interquartile range (IQR). For data not consistently provided across all studies, this approach was adopted to allow for a descriptive comparison across studies, while acknowledging that it may not reflect patient-level variability.

## Results

The search strategy identified 833 studies, of which 12 met the inclusion criteria for the systematic review, covering a total cohort of 445 women. Of these studies, one (8.3%) was prospective, seven (58.3%) retrospective, two (16.7%) were case series and two (16.7%) were case reports. Data regarding physical status and comorbidities were extracted into Table 1, according to the ASA score (American Society of Anesthesiologists) or CCI score (Charlson Comorbidity Index). The data from 445 patients reported a weighted mean age of 52.3 ± 14.2 years. The weighted mean BMI was 26.8 ± 3.0 kg/m². A total of 142 out of 340 women (41.76%) had a history of prior abdominal or pelvic surgery, such data was not homogeneously reported in six studies.

**Table 1:** Population baseline characteristics. (Abbreviations: ASA, American society of Anesthesiology; BMI, body mass index; CCI, Charlson comorbidity Index; ND, not defined)

Year	Authors	Study design	Number of Patients	Age (years mean)	BMI (kg/m2 mean)	Comorbidity (% of patients, ASA, CCI score)
2023	R. G. Olsen et al. [18]	Case series	12	38	23.4	100%, ASA < 2
2023	C. Collà Ruvolo et al. [19]	Retrospective	15	69	26.6	13.3%, ASA 1 80%, ASA 2 6.7%, ASA 3 53.3%, CCI 3
2023	A. Mottaran et al. [22]	Case series	5	68	23.4	ASA 2 (median)
2023	H. Komatsu et al. [23]	Case report	1	40	30.5	ND
2024	Y. Yagur et al. [21]	Retrospective	60	ND	23.7	ND
2024	M. Afonina et al. [15]	Retrospective	32	51	24.2	40.6%, CCI 0 25%, CCI 1 15.6%, CCI 2 18.8%, CCI 3
2024	A. Gioè et al. [16]	Prospective	138	56.0	26.0	ASA 2 (median)
2024	M. Matsuura et al. [25]	Retrospective	10	56	23.7	ASA 1 (median)
2024	K. Law et al. [26]	Case report	1	64	24.2	CCI 0
2024	M. M. Ianieri et al. [17]	Retrospective	16	39	26.6	ND
2024	M. Á. Cáceres Yap et al. [20]	Retrospective	144	49	30.5	24% (hypertension 7.6% and diabetes 2.8%)
2025	D. Kannan Sakthivel et al. [24]	Retrospective	11	ND	26.0	27.3%

Most studies were based on benign gynecologic conditions, with overlapping indications of fibromatosis (*n* = 6, 50%), endometriosis (*n* = 6, 50%), adenomyosis (*n* = 2, 16.7%), pelvic organ prolapse (*n* = 5, 41.7%), and adnexal masses (*n* = 5, 41.7%). Some studies (*n* = 5, 41.7%) also included patients with gynecologic malignancies, particularly endometrial cancer, with one specifically reporting the robotic surgical management of a perivesical recurrence of endometrial cancer. Another study focused on patients diagnosed with vaginal fistulas developed as postoperative or postpartum complications. Hysterectomy was the procedure that was the most performed (either total or supracervical, *n* = 289; 64.9%). None of the included studies reported the use of indocyanine green (ICG) for sentinel lymph node mapping in robotic endometrial cancer procedures.

### Surgical outcomes

Few conversions were reported (*n* = 6, 1.35%), mainly to laparoscopy (*n* = 5, 1.12%), and one (0.22%) to laparotomy. Among these, two laparoscopic conversions were due to console malfunction [15], two additional conversions to laparoscopy were performed for adhesiolysis in case of deep endometriosis by a novice robotic surgeon, and one conversion to laparotomy was reported for the safe management of a 20 cm adnexal mass [16]. Another laparoscopic conversion occurred due to pelvic bleeding, and corresponds to the intraoperative complication described below [17].

Four intraoperative complications (0.90%) across two studies [16, 17] were: two cases of bladder injury during hysterectomy, both managed with sutures and prolonged postoperative catheterization; one retroperitoneal hematoma in the presacral area following transumbilical optic access in a patient with a low BMI; one case of pelvic bleeding requiring the aforementioned conversion to laparoscopy. None of these intraoperative complications were attributed to the robotic system malfunctions. Table 2, and 3 summarize the reported complications/conversions and surgical outcomes, with respect to cohort size, treated disease, type of intervention, docking time, console time, operative time, estimated blood loss, conversion rate, length of hospital stay, and surgical outcome.

**Table 2:** List of reported complications and conversion rate (Abbreviation: CD, Clavien-Dindo, LPS laparoscopy, LPT laparotomy)

Authors	Study design	Number of Patients	Conversion to laparoscopy/laparotomy *n* (%)	Complications *n* (%), CD grade
A. Gioè et al. [16]	Prospective	138	LPT 1 (0.22%) LPS 2 (0.44%)	1 (0.22%) (CD grade I) 3 (0.67%) (CD grade II)
A. Mottaran et al. [22]	Case series	5	None	None
C. Collà Ruvolo et al. [19]	Retrospective	15	None	1 (0.22%) (CD grade I)
D. Kannan Sakthivel et al. [24]	Retrospective	11	None	None
H. Komatsu et al. [23]	Case report	1	None	None
K. Law et al. [26]	Case report	1	None	None
M. Á. Cáceres Yap et al. [20]	Retrospective	144	None	1 (0.22%) (CD grade I)
M. Afonina et al. [15]	Retrospective	32	LPS 2 (0.44%)	2 (0.45%) (CD grades III-IV)
M. M. Ianieri et al. [17]	Retrospective	16	LPS 1 (0.22%	2 (0.45%) (CD grade II)
M. Matsuura et al. [25]	Retrospective	10	None	None
R. G. Olsen et al. [18]	Case series	12	None	4 (0.89%) (CD grade I)
Y. Yagur et al. [21]	Retrospective	60	None	None

**Table 3:** Surgical outcomes (Abbreviation: CSSD, caesarean section scar defect; POP-Q pelvic organ prolapse quantification (POP-Q) System; ND, not defined)

Author		Indication	Procedures	Docking time (minutes, mean)	Console time (minutes, mean)	Operative time (minutes, mean)	Conversion rate (no/yes: *n*./tot (%))	Estimated blood loss (mL, median)	Length of hospital stay (day, mean)
A. Gioè et al. [16]		Pelvic organ prolapse: 60 (43.5%) Fibroids: 43 (31.2%) Adnexal mass: 14 (10.2%) BRCA mutation: 11 (8%) Endometrial hyperplasia: 6 (4.3%) Endometriosis: 2 (1.4%) Endometrial cancer: 2 (1.4%)	Robot-assisted total hysterectomy: 70 (50.7%), Robot-assisted supracervical hysterectomy: 50 (36.2), Sacrocolpopexy, oophorectomy, excision of endometriosis: 18 (13.0)	5.75	119.8	168.3	Yes, 3/138 (3.9%)	216.2	2.7
A. Mottaran et al. [22]		Pelvic organ prolapse (≥ 2-grade POP-Q)	Robot-assisted sacrocolpopexy (RSCP)	ND	91.6	136.6	no	21.6	1.67
C. Collà Ruvolo et al. [19]		Pelvic organ prolapse (≥ 2-grade POP-Q)	Robot-assisted sacrocolpopexy (RSCP)	ND	ND	128.6	no	ND	1.8
D. Kannan Sakthivel et al. [24]		Vesicovaginal fistula (VVF)	Robot-assisted VVF repair	ND	ND	82	no	60	ND
H. Komatsu et al. [23]		Fibromatosis	Robot-assisted total hysterectomy	15	126	177	ND	20	5
K. Law et al. [26]		Endometrial cancer	Robot-assisted total hysterectomy; bilateral salpingo-ophorectomy	10	215	ND	ND	50	4
M. Á. Cáceres Yap et al. [20]		Fibromatosis: 96 (66.7%), Pelvic adhesion syndrome: 29 (20.1%), Endometriosis: 25 (17.4%), Adenomyosis: 24 (16.7%), Benign ovarian mass: 19 (13.2%), Abnormal uterine bleeding: 18 (12.5%), Gynecological cancer: 7 (4.9%)	Hysterectomy: 104 (72.2%), Myomectomy: 20 (13.9%), Oophorectomy: 8 (5.6%)	5.50	ND	118.2	no	43.7	1.78
M. Afonina et al. [15]		Endometrial hyperplasia (9), Leiomyomas (6), Benign ovarian tumors (5), Endometriosis (5), Pelvic organ prolapse (5), Endometrial cancer (2)	Hysterectomy: 20(62.5%), Adnexal surgery: 7 (21.88%), Pelvic floor reconstructive surgery: 5 (15.63%)	8.43	61.8	ND	Yes, 2/32 (6.3%)	42.7	1.33
M. M. Ianieri et al. [17]		Endometriosis	Robot-assisted excision of deep endometriosis	6.36	ND	198	Yes, 1/16 (6.2%)	79.1	3
M. Matsuura et al. [25]		Endometrial cancer: 5 (50.0%) Fibromatosis: 3 (30.0%) Adenomyosis: 2 (20.0%)	Robot-assisted total hysterectomy	ND	ND	112.0	no	5.0	ND
R. G. Olsen et al. [18]		Endometriosis	Unilateral/bilateral salpingo-oophorectomy; ovarian cystectomy; total hysterectomy	ND	ND	ND	no	ND	ND
Y. Yagur et al. [21]		Various benign gynecologic conditions such as endometriosis, fibromatosis, prolapse	Endometriosis total: 32 (53%) (Stages I–II: 24; Stage III–IV: 8) [Segmental bowel resection (1), rectal shaving (2)], Adnexal surgery: 6 (10%), Cerclage: 1 (2%), CSSD repair: 1 (2%), Myomectomy: 3 (5%), Hysterectomy: 16 (27%), Hysterectomy + sacrocolpopexy: 1 (2%)	5.51	65.2	ND	no	ND	ND

All studies reported data on postoperative complications and Clavien-Dindo grade ≥ II complications occurred in 7 (1.57%) out of 445 patients (Table 2). In detail, six minor complications (Clavien-Dindo grade I) included nausea, abdominal pain, transient urinary retention, vaginal bleeding, and mild wound pain (16–18); one case of fever, one urinary tract infection, and two surgical site infections (14, 15); and a single episode of desaturation requiring oxygen therapy (Clavien-Dindo grade II). One patient was readmitted one week after hysterectomy due to unilateral hydronephrosis, necessitating ureteral reimplantation (Clavien-Dindo grade III); another patient from the same cohort was admitted to the intensive care unit (ICU) following hysterectomy due to acute hypercapnia, fully recovered the following day (Clavien-Dindo grade IV) [15].

The time required for surgeries was reported in eleven studies [13–15, 17–24] and divided into docking time, console time and operative time (Table 3). The weighted mean docking time of 392 procedures from seven studies was 5.90 ± 0.94 min [15–17, 20, 21, 23, 26], the weighted mean console time of 237 procedures from six studies was 97.98 ± 28.13 min [13, 14, 19–21, 24], and the weighted mean operative time of 340 procedures was 141.84 ± 28.73 min from eight studies [14, 15, 17, 18, 20–23]. The variability of operative times among different studies reflected the heterogeneity of the procedures. Estimated blood loss (EBL) was reported in nine studies, with a weighted mean of 110.75 ± 84.16 mL [13–15, 18, 20–24]. The weighted mean length of hospital stay was 2 ± 1 days for 352 patients from eight studies [13–15, 17, 18, 20, 21, 24].

### Robotic system

**Fig. 3:**
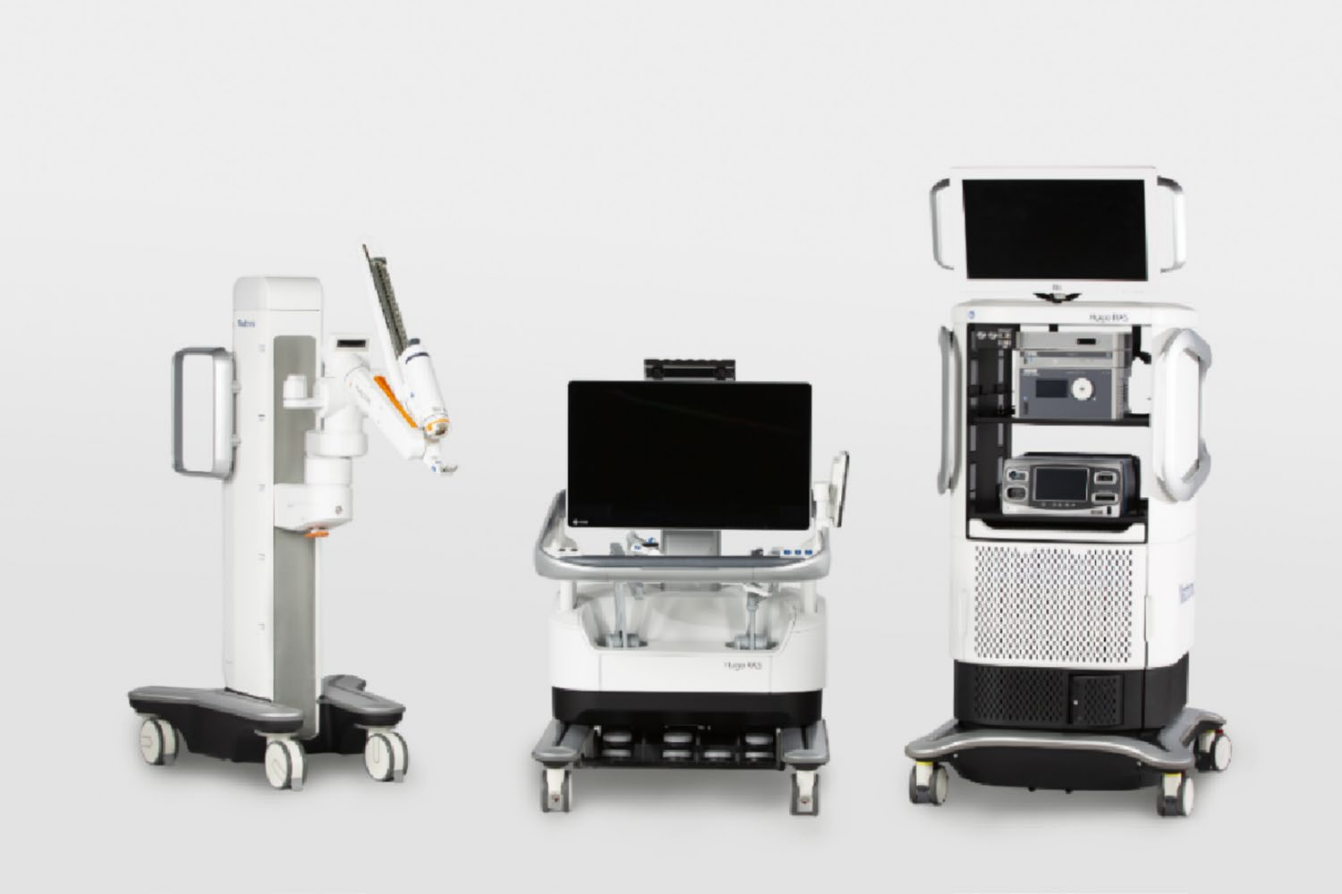
Hugo™ RAS surgical system platform with the surgeon console (center), system tower including the electrosurgical unit (right) and one of the bedside arm carts (left). Image courtesy of Medtronic, reproduced with permission

The Hugo™ RAS comprises an open surgeon console (Fig. 3, center) equipped with a 3D high-definition display. A tracking system monitors the operating surgeon’s glasses and activates a safety lock when visual attention is diverted from the screen. Additional 3D glasses for observers do not interfere with the tracking function. System control is achieved using pistol-grip hand controllers and standard foot pedals. The system tower (Fig. 3, right) contains the core processing units, power management components, backup battery, and imaging and energy systems, including a laparoscopic camera (3D Tipcam™, Karl Storz SE & Co. KG, Tuttlingen, Germany), a high-frequency electrosurgical generator (Valleylab™ FT10 energy platform, Covidien, Dublin, Ireland), and a vessel-sealing system (LigaSure™). The tower serves as the central interface linking the console to the robotic arms, managing power distribution, data processing, and visualization. The platform also includes independent bedside units with one camera cart and up to three instrument carts (Fig. 3, left). Each robotic arm is positioned at a predefined docking angle for a specific procedure setup that can be adjusted according to port placement and patient positioning to optimize instrument alignment and ergonomics [27].

Several studies reported the learning curve associated with the adoption of the Hugo™ RAS system. Docking and console times generally decreased with increasing surgeon experience [15–18, 21]. Conversely, one study noted a reduction in docking time with staff efficiency over time, while console time remained largely unchanged [24]. Some investigations involving surgeons with prior experience on other robotic platforms reported a steep and short learning curve for console times, likely attributable to skills transferability between systems [18, 19]. Other observations highlighted iterative improvements in docking efficiency achieved through modifications in port placement [23].

Various arm configurations were explored to minimize instrument collisions. In the field of pelvic surgery, both “straight” and “bridge” port placement options have been described, whereas the so-called “compact” and “butterfly” configurations refer to the positioning of the arm carts [28]. However, the application of these configurations and their minor modifications tend to vary across surgical teams, depending on institutional practice and specific procedural requirements.

A total of 34 system errors over 138 procedures related to instrument and system malfunctions were reported in one study [16]. Among these, robotic arm malfunctions were the most frequent ones (61.8%), followed by instrument failures (26.4%) and console errors (11.8%). A 24-minute procedural pause during a robot-assisted total hysterectomy was reported due to unspecified equipment issues [23]. Other studies reported minor calibration and tracking anomalies necessitating intraoperative component replacement, which adversely affected surgical workflow efficiency but did not compromise patient safety [18, 21]. Some studies [19, 20, 26] reported occasional instrument malfunctions that did not significantly affect the conduct or outcome of the surgical procedures. Certain console errors were resolved without patient complications or conversion to alternative surgical approaches [16, 18]. In contrast, console malfunction required two conversions to laparoscopy, managed successfully by continuing to use the robotic platform’s integrated tower [15].

## Discussion

This review presents a comprehensive summary of the available studies on the use of the Hugo™ RAS system in gynecologic surgery. Total or supracervical hysterectomy was the procedure that was the most commonly performed. The conversion rate was low, and intra- and postoperative complications were rare, representative of a safe adoption of a novel surgical approach in the field of minimally invasive gynecologic surgery. No bowel injuries were observed, in line with the reported low incidence in robotic gynecologic surgery [29].

The first European gynecologic procedure with the Hugo™ RAS system was a total hysterectomy and salpingo-oophorectomy performed by Professor Scambia and his team in a woman affected by BRCA-1 mutation in 2022 [9]. In 2023, the first case series of robotic sacral colpopexies (RSCPs) followed, suggesting its effectiveness with minimal intraoperative and postoperative complications [6]. Its feasibility of performing surgical interventions for endometriosis was also investigated, including complex procedures for deep intestinal endometriosis [5, 15, 25, 26].

The Hugo RAS system introduced features such as the open surgical console, equipped with a glasses-based safety monitoring system that tracks the surgeon’s head position and gaze direction. This design was reported to guarantee both safety and communication with the operating room team in initial clinical experiences [28]; conversely, other surgeons reported preference for closed consoles, citing greater focus and comfort [31]. The setup of independent bedside units, customized with different tilt angles to suit the patients’ body type and surgeons’ preferences, was reported to require more operative space when compared to so-called monolithic (single-boom) bedside units [6].

One retrospective observational study compared the use of the da Vinci robotic surgical system to the Hugo™ RAS system in endometriosis surgery [17]. Six postoperative complications were reported (20% in the da Vinci group, 12.5% in the Hugo™ RAS group), with no significant differences in operative time, blood loss or hospital stay. The longer docking times for the Hugo™ RAS were attributed to its multi-arm design and the initial learning curve of the surgical team.

In general, docking duration correlated inversely with the team’s level of experience and typically decreased with proficiency [32]. Of note, this phenomenon was corroborated by several studies, as well as incremental gains in efficiency through refinements in port placement, made possible by the system’s versatile configuration options to minimize arm interference and resolve issues [23].

None of the included studies specifically examined the impact of general robotic surgery training or a dedicated training with the newly adopted platform on surgical outcomes. Nonetheless, several reports indicated that participating surgeons had prior experience with the da Vinci system and underwent a structured Hugo™ RAS training program, combining theoretical instructions, simulator-based exercises and hands-on practice [15, 17–21]. These data underscore the potential for smooth transition of surgeon’s skills to the new robotic platform.

Concerning gynecologic malignancies, the current literature supports minimally invasive surgery in select patient populations [33]. Specifically, robotic surgery offers advantages for severely obese patients by supporting abdominal weight and allowing lower intra-abdominal pressure during Trendelenburg positions [34, 35]. The ongoing prospective randomized ROBESE trial is directly comparing laparoscopic versus robotic approaches in obese patients with early-stage endometrial cancer, focusing on conversion rates [36]. For early-stage cervical cancer, the LACC trial established laparotomy as a standard of care after finding higher recurrence rates and poorer overall survival with minimally invasive surgery [37]. To provide further clarity on this issue, two ongoing randomized controlled trials are underway to assess the oncological safety of robotic versus open surgery [38, 39].

In this systematic review, four research groups reported endometrial cancer management using the Hugo™ RAS system [15, 16, 25, 26]. One reported its use for the treatment of stage IA endometrial cancers in which no lymph node dissection was performed [25]. A robotic staging procedure with pelvic and para-aortic lymphadenectomy was described [26], noting no major complications and efficient upper abdominal access through arm angle adjustments without redocking. Successful management of isolated paravesical endometrial cancer recurrences was also documented [16]. However, among these studies, information regarding the use of sentinel lymph node (SLN) dissection for staging was missing, with no documented use of indocyanine green for SLN mapping.

Overall, the available literature demonstrated effective adoption of the Hugo™ RAS system in gynecologic surgery, with consistent use across a range of procedures during initial experiences. As with any new technology, safe implementation relies on dedicated training for surgeons and operating room teams and adequate patient selection to maximize its potential benefits.

### Study limitations

The primary limitation of the present systematic review stems from the recency of the system’s introduction, with still limited and heterogeneous data over 3 years of increasing adoption in gynecologic surgery, primarily comprising retrospective studies, pilot studies, case series and case reports with small cohort sizes and short follow-up periods. Publication bias cannot be ruled out, as smaller-scale or negative experiences may remain unpublished during this initial implementation phase. Consequently, even if functional outcome findings appear promising, summary estimates should be interpreted with caution, and further research with larger cohort and extended follow-up is necessary to enhance reliability. Additionally, the inclusion of early case series may affect the results due to the impact of the learning curve. Furthermore, included studies did not report detailed data on learning curves depending on the number of procedures or training duration, preventing assessment of outcomes relative to early experience or proficiency stages. The recent introduction of indocyanine green (ICG) fluorescence is expected to broaden patient recruitment, particularly for endometrial and cervical cancers with SLN approaches, hence facilitating a more comprehensive analysis of long-term oncological outcomes.

### Implications for practice and future research

Beyond technical advantages, such as tremor suppression, reduced musculoskeletal strain and lower cognitive workload for surgeons compared with conventional laparoscopy [40], the advantage of robotic surgery lies in its integration with emerging digital technologies [8].

This review provides an overview of the current surgical approaches and outcomes associated with Hugo™ RAS in gynecology, with early clinical evidence showing suitability for gynecologic indications. However, the next step is to look beyond standard perioperative endpoints and objectively assess the benefits of integrating new technologies into robotic-assisted surgery.

Compared to laparoscopy, robotic surgical workflow analysis, through deep learning (DL) algorithms applied to surgical video data, appears easier, and further computer vision studies are underway to enhance the safety of procedures [8, 41], including with Touch Surgery™ ecosystem (Medtronic, Minneapolis, MN, United States). Future research should not only compare surgical outcomes but also capture how technology integration may enhance workflow efficiency, safety and ultimately value of care. The lack of tactile feedback in robotic surgery is increasingly offset by instrument articulation and force feedback, high-resolution and fluorescence imaging, augmented reality systems [42, 43] and modern tissue assessment technologies, such as high-frequency (up to 70 MHz) and ultra-high-frequency (up to 100 MHz) ultrasound probes. Along with innovative optical imaging systems, they are set to improve real-time intraoperative tissue analysis beyond the tactile capacities of human fingers [42–45].

The evolution of robotic surgery towards telesurgery with multi-console integration [48] expands the possibilities of remote collaboration and holds the potential to advance tele-image-guided surgery by delivering timely expert care to patients requiring urgent or specialized imaging, in settings where local access is limited [49]. As demonstrated during the Society of Robotic Surgery 2025 Congress at the IRCAD Research and Training Center in Strasbourg, France, the Hugo™ RAS system remote telesurgery capabilities can support long-distance collaboration, and further connectivity via smart assistance tools.

Proficiency in robotic surgery requires intensive, sustained training [50]. Recognizing the need for validated training curricula, two recent investigations [50, 51] assessed the efficacy of advanced robotic training using the Hugo™ RAS simulator in skills improvements among novice trainees from various specialties, thereby suggesting a step towards the development of standardized programs. Systematic surgeon-led training in RAS is further complicated by the diversity of robotic platforms. Evaluation of new robotic systems should follow the principles of the IDEAL framework, but it does not directly address the challenge of comparing outcomes between two conceptually similar robotic platforms, ideally with the inclusion of a laparoscopic control arm [52]. Only surgeons who have completed the learning curve in each arm would be eligible for comparative trials, as surgical experience is an important source of variability and bias [52]. High-quality studies should be designed to capture these factors in their methodology and analysis.

Although patient outcomes are likely to be similar across platforms with surgeons driven to safely adopt new technology, differences may emerge in terms of cost. Thorough research into cost-effectiveness and accessibility is still needed to better evaluate the impact of robotic-assisted surgery on the healthcare system. Early financial analyses in urologic oncology demonstrated an approximately 11% reduction in direct per-procedure costs for radical prostatectomies performed with the Hugo™ RAS system compared with those performed using the da Vinci platform, though limited by small sample size [53]. In contrast, a large-scale analysis demonstrated that, overall, robotic-assisted procedures remain more expensive than laparoscopic surgeries, regardless of the type of intervention [54]. Nevertheless, compared to laparoscopic procedures, robotic procedures were associated with a significant 2.2% lower complication rate and a 0.7-day shorter hospital stay [54]. Future gynecologic investigations should incorporate cost-effectiveness evaluation, both in comparison to standard laparoscopy and to various robotic platforms, within a value-based healthcare framework, to assess the potential for implementation, even within resource-constrained healthcare environments.

## Conclusions

The available evidence appears to represent a safe and effective use of the Hugo™ RAS system for a wide range of gynecologic procedures. Despite areas requiring further optimization, the system appeared to perform reliably, without major intraoperative complications reported. As surgical experience with the Hugo™ RAS system expands, ongoing research will aid to refine its integration with new digital technologies into standard gynecologic practice.

## Supplementary Information

Below is the link to the electronic supplementary material.

Supplementary Material 1

Supplementary Material 2

## Data Availability

All data generated or analysed in this review are included in this article and/or its figures. Further enquiries can be directed to the corresponding author.
