# Supplementary material for: The Hugo™ RAS system in gynecologic robotic surgery: a systematic review of current applications
Source: J Robot Surg. 2025 Nov 17;20(1):22. doi: 10.1007/s11701-025-02973-3 (PMC12620324; doi:10.1007/s11701-025-02973-3)
Supplement: Supplementary file 1 — Supplementary Material 1 [file 11701_2025_2973_MOESM1_ESM.docx]

| **Databases** | **Search strategy** |
| --- | --- |
| (HUGORAS OR Hugo OR HUGOTM OR medtronic robotic OR medtronic robot) AND (system OR RAS OR surgery OR surgical OR gynecology OR urology OR endocrine OR digestive OR bariatric OR training OR artificial intelligence) | |
| MEDLINE ® | (HUGORAS[Title/Abstract] OR Hugo[Title/Abstract] OR HUGOTM[Title/Abstract] OR medtronic robotic[Title/Abstract] OR medtronic robot[Title/Abstract]) AND (system[Title/Abstract] OR RAS[Title/Abstract] OR surgery[Title/Abstract] OR surgical[Title/Abstract] OR gynecology[Title/Abstract] OR urology[Title/Abstract] OR endocrine[Title/Abstract] OR digestive[Title/Abstract] OR bariatric[Title/Abstract] OR training[Title/Abstract] OR artificial intelligence[Title/Abstract]) **Field: title OR abstract** |
| EMBASE ® | (hugoras:ti,ab,kw OR hugo:ti,ab,kw OR hugotm:ti,ab,kw OR 'medtronic robotic':ti,ab,kw OR 'medtronic robot':ti,ab,kw) AND (system:ti,ab,kw OR ras:ti,ab,kw OR surgery:ti,ab,kw OR surgical:ti,ab,kw OR gynecology:ti,ab,kw OR urology:ti,ab,kw OR endocrine:ti,ab,kw OR digestive:ti,ab,kw OR bariatric:ti,ab,kw OR training:ti,ab,kw OR 'artificial intelligence':ti,ab,kw) AND [2019-2024]/py **Field: title OR abstract OR keywords** |
| Web of science ™ | ((TI=(Databases (HUGORAS OR Hugo OR HUGOTM OR medtronic robotic OR medtronic robot) AND (system OR RAS OR surgery OR surgical OR gynecology OR urology OR endocrine OR digestive OR bariatric OR training OR artificial intelligence))) OR AB=(Databases (HUGORAS OR Hugo OR HUGOTM OR medtronic robotic OR medtronic robot) AND (system OR RAS OR surgery OR surgical OR gynecology OR urology OR endocrine OR digestive OR bariatric OR training OR artificial intelligence))) OR KP=(Databases (HUGORAS OR Hugo OR HUGOTM OR medtronic robotic OR medtronic robot) AND (system OR RAS OR surgery OR surgical OR gynecology OR urology OR endocrine OR digestive OR bariatric OR training OR artificial intelligence)) **Field: title OR abstract OR keywords** |
