# Supplementary material for: The Hugo™ RAS system in gynecologic robotic surgery: a systematic review of current applications
Source: J Robot Surg. 2025 Nov 17;20(1):22. doi: 10.1007/s11701-025-02973-3 (PMC12620324; doi:10.1007/s11701-025-02973-3)
Supplement: Supplementary file 2 — Supplementary Material 2 [file 11701_2025_2973_MOESM2_ESM.docx]

**Excluded studies**

| **YEAR** | **AUTHORS** | **ARTICLE** | **EXCLUSION-REASONS** | **PMID** |
| --- | --- | --- | --- | --- |
| 2022 | G. Monterossi | The first European gynaecological procedure with the new  surgical robot HugoTM RAS. A total hysterectomy and salpingo-oophorectomy in a woman affected by BRCA-1 mutation | data already included in other studies | 35373554 |
| 2023 | G. Campagna | Robotic sacrocolpopexy plus ventral rectopexy as combined treatment for multicompartment pelvic organ prolapse using the new Hugo RAS system | data already included in other studies | 36786846 |
| 2023 | G. Panico | The first 60 cases of robotic sacrocolpopexy with the novel HUGO RAS system: feasibility, setting and perioperative Outcomes | data already included in other studies | 37215346 |
| 2023 | G. Monterossi | The new surgical robot HugoTM RAS for total hysterectomy:  a pilot study | data already included in other studies | 38128091 |
| 2023 | M. Pavone | En-block butterfly excision of posterior compartment deep  endometriosis: The first experience with the new surgical robot HugoTM RAS | data already included in other studies | 38128095 |
| 2024 | D. Kannan | Robotic Surgery in Pelvic Organ Prolapse: A  Retrospective Comparison of Ileopectopexy and  Sacrocolpopexy | wrong study design | 39583429 |
| 2024 | M. Pavone | Initial experience of robotically assisted endometriosis surgery with a novel robotic system: first case series in a tertiary care center | data already included in other studies | 38133880 |
